# Dihydroporphyrin iron (III) enhances low temperature tolerance by increasing carbon and nitrogen metabolism in *Andrographis paniculata*

**DOI:** 10.3389/fpls.2024.1522481

**Published:** 2025-01-03

**Authors:** Shao-Fen Jian, Yan-Fen Huang, Dong-Liang Chen, Chu Zhong

**Affiliations:** ^1^ National Center for Traditional Chinese Medicine (TCM) Inheritance and Innovation, Guangxi Botanical Garden of Medicinal Plants, Nanning, China; ^2^ Guangxi Key Laboratory of Medicinal Resource Protection and Genetic Improvement, Guangxi Botanical Garden of Medicinal Plants, Nanning, China; ^3^ Guangxi Engineering Research Centre of Traditional Chinese Medicine (TCM) Intelligent Creation, Guangxi Botanical Garden of Medicinal Plants, Nanning, China; ^4^ Cash Crops Research Institute, Guangxi Academy of Agricultural Sciences, Nanning, China

**Keywords:** dihydroporphyrin iron, low temperature stress, carbon metabolism, *Andrographis paniculata*, antioxidant

## Abstract

Dihydroporphyrin iron (DH-Fe) is a novel plant growth regulator that plays significant roles in plant stress resistance. We found that *Andrographis paniculata* is extremely sensitive to low temperature (LT) with a threshold of 25°C. To evaluate whether and how DH-Fe alleviates LT stress in *A. paniculata*, different DH-Fe concentrations (0, 10, 20, and 40 μg·L^-1^) were applied to estimate its effects on C and N metabolism and antioxidative capacity in *A. paniculata* grown under 20°C. Pre-treatment of DH-Fe alleviated LT-induced anthocyanin accumulation. Additionally, it relieved LT-induced oxidative stress by increasing the activity of catalase (CAT). DH-Fe reduced the contents of sucrose, soluble sugar and starch and the activities of sucrose synthase (SS) and hexokinase (HXK), but stimulated the activities of sucrose phosphate synthase (SPS), glucose-6-phosphate dehydrogenase (G6PDH), glyceraldehyde 3-phosphate dehydrogenase (GAPDH), phosphoenolpyruvate carboxylase (PEPC), isocitrate dehydrogenase (ICDH), and malic enzyme (ME). Soluble protein and proline contents were decreased by DH-Fe, while total N and free amino acids contents were increased, accompanying by the enhancement of the activities of glutamine synthase (GS), glutamic-oxaloacetic transaminase (GOT) and glutamic-pyruvic transaminase (GTP). Simultaneously, the content of andrographolide, the bioactive ingredient of *A. paniculata*, was remarkably declined. These results indicated that DH-Fe alleviates LT-induced oxidation by increasing sugar catabolism and allocating C metabolic flow to N assimilation. A concentration of 20 μg·L^-1^ DH-Fe is recommended to be used to enhance LT tolerance in *A. paniculata*. Our results update the understanding of the mechanism of plant cold tolerance and provide new ideas for relieving plant cold damage.

## Highlights

- *Andrographis paniculata* is sensitive to low temperature with a threshold of 25°C.
- Low temperature stress increases sugar accumulation but decreases nitrogen accumulation in *A. paniculata*.
- DH-Fe enhances the activities of carbohydrate metabolism and nitrogen assimilation enzymes.
- DH-Fe alleviates oxidative stress of plants under low temperature.

## 1 Introduction

Temperature is a key environmental factor that determines plant growth and distribution. The frequent occurrence of extreme weather due to climate change and the introduction of plants in higher latitude areas have resulted in short-term low temperature (LT) stress on plants. LT stress is divided into chilling stress (< 20°C) and freezing stress (< 0°C) according to the environmental temperature. The underlying mechanisms by which LT constrains plant growth and productivity are complex (Ding et al., 2019). The most common impact of LT stress on plants is disruption of the integrity of plant cell membranes, thereby increasing oxidative stress and inhibiting plant growth (Manasa et al., 2022).

Since plants are sessile, they adapt to environmental fluctuations through metabolism. There are many physiological protective mechanisms for plants to cope with LT stress. Plants can enhance osmotic regulation ability through accumulation of osmotic substances such as soluble sugars and proline (Raza et al., 2023a), and increase reactive oxygen species (ROS) scavenging capacity through enzymatic and non-enzymatic pathways to maintain cell redox homeostasis (Kocsy et al., 2001).

Carbon (C) and nitrogen (N) metabolisms are of great importance in plant adaptation to abiotic stresses (Cui et al., 2019). Exposure to LT stress usually causes a decline in growth and N assimilation, but increases carbohydrate accumulation. Sucrose catabolism plays important roles in alleviating plant stress (Zhang et al., 2024). C partitioning and the generation of reductants (NADPH) catalyzed by dehydrogenases protect plants from LT stress (Airaki et al., 2012; Kitashova et al., 2023). It has also been widely reported that enhanced N metabolism increases plant fitness under the stresses such as drought (Zhong et al., 2019), salinity (Shao et al., 2015), heavy metal (Hussain et al., 2020), and high and low temperature (Luo et al., 2023; Soualiou et al., 2023). Therefore, modulating carbon and nitrogen metabolism could ameliorate plant stress tolerance.

Exogenous application of plant growth regulator plays an important role in alleviating plant LT stress and restoring plant productivity (Zhou et al., 2003; Ritonga and Chen, 2020). For examples, exogenous 5-aminolevulinic acid (ALA) protects plants against cold-triggered oxidative stress by interacting with GABA, NO, H_2_O_2_, and JA (Liu et al., 2018, 2019, 2020); melatonin alleviates plants cold stress by regulating redox homeostasis (Li et al., 2018a). Some plant hormones and analogue such as abscisic acid, salicylic acid, and trinexapac-ethyl also act as growth regulators which can enhance plant osmotic regulation and antioxidant capacity (Guan et al., 2018; Raza et al., 2023b). Cao et al. (2017) reported that exogenous glycine alleviates cold stress of rice plant by mediating N uptake, photosynthesis, and antioxidant defense ability. Dihydroporphyrin iron (DH-Fe) is a novel plant growth regulator derived from silkworm excrement. Due to its characteristics of safety and green and pollution-free, it has been extensively used in enhancing crop stress resistance and improving the yield of cereal and economic crops (Cao et al., 2016, 2018; Chu et al., 2023; Yang et al., 2023). It is consistently revealed that DH-Fe ameliorates plant photosynthesis and antioxidant capacity. However, the effects of DH-Fe on plant LT stress resistance and the physiological mechanism have not been well understood.

*Andrographis paniculata* is a medicinal plant originating from tropical regions that could be sensitive to LT stress. It has been introduced to and mainly cultivated in the south China since the early 20^th^ century. With the growing demand of pharmaceutical industry for the herb, it has been recently introduced northward to the area north of the Yangtze River, where the temperature could be the key factor restricting the growth and the completion of its life cycle. Given the excellent performance of DH-Fe in ameliorating plant stress resistance, this study intended to investigate the protective effect of DH-Fe on *A. paniculata* under LT stress. We aimed to reveal the physiological mechanism by which DH-Fe rescues *A. paniculata* plants from LT stress. It could provide new ideas for relieving plant cold damage.

## 2 Materials and methods

### 2.1 Plant materials and treatments

*Andrographis paniculata* (Burm. f.) Ness from the Acanthaceae was used as experimental material. The seeds were provided by the seed bank of Guangxi Botanical Garden of Medicinal Plants. Genetic stable and consistent lines were obtained by single plant selection and cultivation for three generations. The seeds were sown on seedbed filled with wet perlite-vermiculite (V: V = 4: 1) and incubated in a growth chamber for germination. The photoperiod was 14 h and light intensity was 100 μmol·m^-2^·s^-1^ provided by LED with a constant temperature of 28°C. Two weeks after germination, the seedlings were transplanted to a seedling-raising plate and continued to grow for a month in the light and temperature conditions described above. The mixture of perlite, vermiculite and organic fertilizer (V: V: V = 4: 1: 2) was used as growth substratum. When the seedlings were 6-leaf age, they were transplanted to pots and grown in the mixture of perlite, vermiculite and organic fertilizer (V: V: V = 4: 1: 2) with the light and temperature conditions above. The seedlings at 12-leaf age were used for temperature treatments. The plants were put in light incubator at constant temperature of 10°C, 15°C, 20°C, 25°C, or 30°C with a photoperiod of 14/10 h light/dark. The intensity of light in the incubator was 100 μmol·m^-2^·s^-1^. The plants were watered once a week with the nutrition solution as reported previously (Huang et al., 2023), to avoid water stress.

DH-Fe was provided by Nanjing Bostec Biological Engineering Co., Ltd. (Nanjing, China), and the content of active ingredient of the product is 0.02%. The concentration of DH-Fe applied in this study was 0, 10, 20, and 40 μg·L^-1^. Plants grew in optimal temperature (30°C) were foliar sprayed with DH-Fe 3 days before LT stress (20°C) treatment. Then the plants were exposed to 20°C and grown in incubator with the light intensity of 100 μmol·m^-2^·s^-1^ and the photoperiod of 14/10 h light/dark. After treatment for 7 days, the plants were sampled for evaluating the effects of DH-Fe on plant LT stress tolerance.

### 2.2 Measurements of total N

Aboveground of the plants were harvested and oven-dried at 70°C to a constant weight. Then the samples were fine pulverized, and about 50 mg powder was used for total nitrogen measurement. The samples were digested with H_2_SO_4_-H_2_O_2_ at 260°C. The contents of NH_4_^+^ and NO_3_^-^ in the digestion solution were measured using the indophenol blue colorimetry method at 625 nm (Novamsky et al., 1974) and ultraviolet spectrophotometry at 210 nm (Norman and Stucki, 1981) with (NH_4_)_2_SO_4_ and KNO_3_ as standard, respectively. Total N content is the sum of NH_4_^+^ and NO_3_^-^ contents.

### 2.3 Anthocyanin measurements

Anthocyanin in fresh leaves was extracted by immersing the samples in acid ethanol solution (0.1 mol·L^-1^ HCl in 95% ethanol) and incubating at 60°C water bath for 1 h. The absorbance of the extract was measured at 530 nm, 620 nm, and 650 nm, respectively. Anthocyanin content based on fresh weight was calculated using the molar extinction coefficient of 4.62 × 10^4^ mM^-1^·cm^-1^ (Sun et al., 2018).

### 2.4 Measurement of hydrogen peroxide and lipid peroxidation

H_2_O_2_ was measured by colorimetry with titanium (Brennan and Frenkel, 1977). Frozen leaf samples (0.1 g) were homogenized with 10 mmol·L^-1^ 3-amino-1,2,4-triazole and centrifuged at 4°C and 9400 g for 10 min. The supernatant was reacted with 0.1% titanium sulphate in 20% H_2_SO_4_. Removing precipitant, the supernatant was measured colorimetrically at 410 nm. H_2_O_2_ was used as standard.

Malondialdehyde (MDA) content represents lipid peroxidation. MDA in leaf samples was prepared as soluble protein described latter. The supernatant was reacted with 0.6% thiobarbituric acid in 5% trichloroacetic acid in boiling water bath for 15 min. After centrifuging at 9400 g for 10 min, the supernatant was measured spectrophotometrically at 450 nm, 532 nm, and 600 nm, respectively. The MDA content was calculated as in Kramer et al (1991) using the coefficient of 0.155 mM^-1^·cm^-1^.

### 2.5 Soluble protein and antioxidant indexes

Soluble protein was extracted by 100 mmol·L^-1^ sodium phosphate buffer (pH7.5) by homogenizing frozen samples in pre-cooled mortar and pestle. The homogenates were centrifuged at 4°C and 9400 g for 10 min, and soluble protein content in the supernatant was measured spectrophotometrically at 562 nm using the BCA-protein assay (Smith et al., 1985).

Catalase (CAT) activity was measured by monitoring the decline rate of absorbance at 240 nm (A_240_) after adding 0.1 mL enzymes extract in 1 mL reaction system containing 50 mmol·L^-1^ sodium phosphate buffer (pH7.8) and 20 mmol·L^-1^ H_2_O_2_. The activity of CAT was represented as μmol H_2_O_2_·mg^-1^ protein·min^-1^. The extinction coefficient of H_2_O_2_ is 36 mM^-1^·cm^-1^ (Havir and Mchale, 1987). Superoxide dismutase (SOD) activity was measured by monitoring the inhibition of photochemical reduction of nitro-blue tetazolium (NBT) (Jalali-e-Emam et al., 2011).

### 2.6 Amino acids and proline

Total free amino acids were measured by ninhydrin method (Sun et al., 2006) with some modifications. Frozen leaf samples (0.1 g) were homogenized with 10% acetic acid, and then centrifuged at 4°C and 9400 g for 10 min. The supernatant was incubated in boiling water bath for 15 min, and then reacted with 0.5% ninhydrin reagent in the mixture of N-propyl alcohol, N-butanol, ethylene glycol, and sodium acetate buffer (pH5.4) (V: V: V: V = 1: 2: 4: 0.6) in boiling water bath for 15 min. The absorbance at 580 nm was detected and the content of amino acid N was calculated with leucine as standard.

Proline in frozen leaf samples (0.1 g) was extracted with 3% sulfosalicylic acid in boiling water bath for 10 min. After cooling, 2 mL extract was reacted with 3 mL 2.5% ninhydrin in the mixture of acetic acid and 6 mol·L^-1^ orthophosphoric acid (H_3_PO_4_) (V: V = 3: 2) in boiling water bath for 40 min. Cooling again, the proline in the solution was extracted with 2 mL methylbenzene, and then measured spectrophotometrically at 520 nm (Bates et al., 1973). The proline concentration was calculated by the standard curve.

### 2.7 Nitrogen metabolism enzymes

Frozen leaf samples (0.1 g) were homogenized with 3 ml 50 mmol·L^-1^ Tris-HCl (pH8.0, containing 2 mmol·L^-1^ Mg^2+^, 2 mmol·L^-1^ DTT and 0.4 mol·L^-1^ sucrose) and centrifuged at 4°C and 9400 g for 10 min. The supernatant was used for measurement of glutamine synthase (GS), glutamate synthase (GOGAT), glutamate dehydrogenase (GDH), glutamic-oxaloacetic transaminase (GOT), and glutamic-pyruvic transaminase (GPT) activities as reported previously (Zhong et al., 2017). Soluble protein in enzyme extract was measured using the BCA-protein assay.

### 2.8 Sugars and starch

Sucrose and soluble sugar were extracted by deionized water at 80°C water bath for 30 min and 3 times. The extracts were collected. Starch in the residues was extracted using perchloric acid after starch was gelatinized at boiling water bath. Sucrose was quantified colorimetrically at 500 nm by the dioxybenzene method (Li et al., 2012), and soluble sugar and starch were measured using the sulfuric acid-anthrone method (Wang et al., 2002). Sucrose and glucose were used as standard.

### 2.9 Quantification of carbohydrate metabolic enzymes activities

Enzymes were extracted by homogenizing frozen leaf samples (~ 0.1 g) with HEPES-KOH (pH7.5) containing 50 mmol·L^-1^ MgCl_2_, 10 mmol·L^-1^ β-mercaptoethanol, 2 mmol·L^-1^ EDTA, and 2% (W/V) PVP. The homogenates were centrifuged at 4°C and 9400 g for 10 min. The supernatant was used for enzyme activity measurements. Sucrose synthase (SS) and sucrose phosphate synthase (SPS) activities were assayed by the kits (Suzhou Michy Biomedical Technology Co., Ltd).

The assay mixture of hexokinase (HXK) contained 100 mmol·L^-1^ Tris-HCl (pH7.5), 2 mmol·L^-1^ MgCl_2_, 10 mmol·L^-1^ KCl, 1 mmol·L^-1^ NAD^+^, 1 mmol·L^-1^ ATP, 1 unit G6PDH, 2 mmol·L^-1^ glucose, and 0.1 mL enzyme extract in a total volume of 1 mL (Dai et al., 1999).

The reaction medium of glucose-6-phosphate dehydrogenase (G6PDH) contained 100 mmol·L^-1^ Tris-HCl (pH8.0), 10 mmol·L^-1^ MgCl_2_, 1 mmol·L^-1^ NADP^+^, 5 mmol·L^-1^ glucose 6-phosphate, and 0.1 mL enzyme extract in a total volume of 1 mL (Airaki et al., 2012).

The assay mixture of glyceraldehyde 3-phosphate dehydrogenase (GAPDH) contained 100 mmol·L^-1^ Tris-HCl (pH8.4), 10 mmol·L^-1^ MgCl_2_, 2.5 mmol·L^-1^ DTT, 5 mmol·L^-1^ ATP, 5 mmol·L^-1^ 3-PGA, 0.2 mmol·L^-1^ NADPH, and 0.1 mL enzyme extract in a total volume of 1 mL (Schulman and Gibbs, 1968).

The phospho*enol*pyruvate carboxylase (PEPE) activity was assayed in a system containing, in a total volume of 1 mL, 100 mmol·L^-1^ Tris-HCl (pH8.0), 10 mmol·L^-1^ MgCl_2_, 25 mmol·L^-1^ NaHCO_3_, 1 mmol·L^-1^ DTT, 0.2 mmol·L^-1^ NADH, 24 units of l-malate dehydrogenase, 8 mmol·L^-1^ PEP, and 0.1 mL enzyme extract (Petersen et al., 2001).

The assay mixture of malic enzyme (ME) contained 100 mmol·L^-1^ HEPES-KOH (pH 7.5), 5 mmol·L^-1^ MgSO_4_, 0.5 mmol·L^-1^ NADP^+^, 5 mmol·L^-1^ l(+)-malate, and 0.1 mL of enzyme extract in a total volume of 1 mL (Kulichikhin et al., 2009).

The assay mixture of isocitrate dehydrogenase (ICDH) contained 100 mmol·L^-1^ K_2_HPO_4_-KH_2_PO_4_ (pH7.5), 50 mmol·L^-1^ MgCl_2_, 1 mmol·L^-1^ NADP^+^, 30 mmol·L^-1^ isocitrate, and 0.1 mL enzyme extract in a total volume of 1 mL (Airaki et al., 2012).

Enzyme activities were calculated by the average rate of NAD(P)^+^ reduction or NAD(P)H oxidation during the first 2 min after the start of the reaction. Soluble protein in enzyme extract was measured using the BCA-protein assay.

### 2.10 Quantification of diterpenoid lactones

Diterpene lactones (andrographolide, neoandrographolide, 14-deoxyandrographolide, and 14-deoxy-11, 12-didehydroandrographolide) were quantified by the HPLC method. About 0.1 g fine pulverized dry leaf samples were immersed in 5 mL 40% (V/V) methanol, and ultrasound extracted for 30 min. Then the extract was filtered by 0.22 μm water-phase filter membrane, and detected by the HPLC (2030C-Plus, Shimadzu, Japan) at 205 nm using the Agilent C_18_ chromatography column with octadecylsilane bonded silica gel as filler. Mobile phase A was acetonitrile, and mobile B was water. Elution gradient: 0-15 min, 80%-75% B and 20%-25% A; 15-30 min: 75%-72% B and 25%-28% A; 30-60 min: 72%-60% B and 28%-40% A; 60-65 min: 60%-15% B and 40%-85% A; 65-70 min, 15%-0% B and 85%-100% A. The injection volume was 10 μL. Column temperature was 30°C, and flow rate was 1 mL·min^-1^.

### 2.11 Statistical analysis

All the tests were carried out in triplicate or quadruplicate, and the results were presented as mean value ± standard deviation. Analysis of significant differences and correlation was performed in SPSS Statistics 19 software (IBM Inc., Chicago, IL, USA), using the one-way ANOVA and Duncan’s multiple range method and Pearson correlation coefficient method, respectively. Statistical significance was considered when *P* < 0.05.

## 3 Results

### 3.1 Low temperature inhibits *A. paniculata* growth and promote oxidation stress

**Figure 1:**
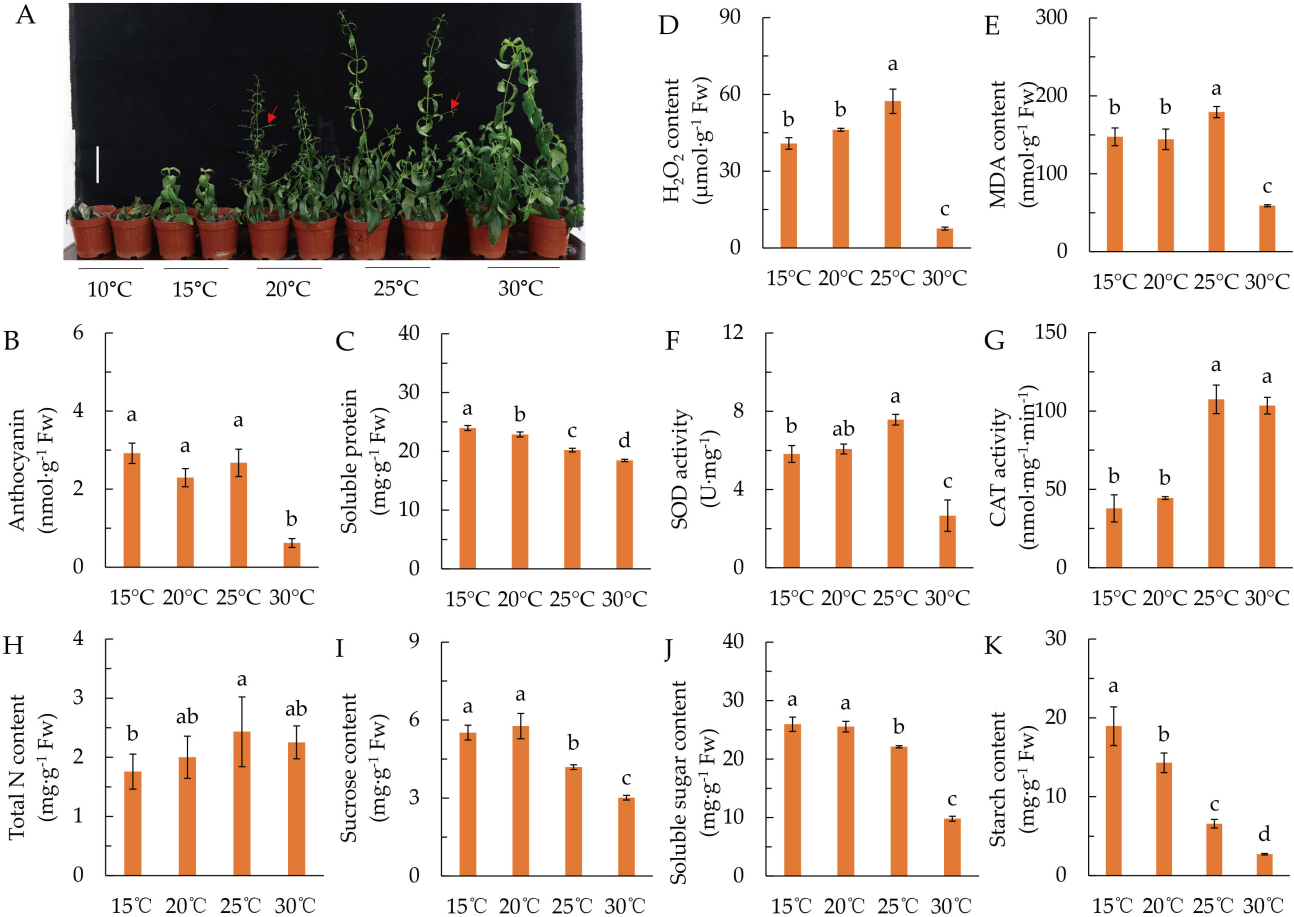
Effects of temperature on plant growth, antioxidant, and the accumulation of nitrogen and sugars. **(A)** Phenotype of plants grown under different temperature for 60 days. Red arrows indicate flower branches. **(B)** Anthocyanin content. **(C)** Soluble protein content. **(D)** H_2_O_2_ content. **(E)** Malondialdehyde (MDA) content. **(F)** Superoxide dismutase (SOD) activity. **(G)** Catalase (CAT) activity. **(H)** Total leaf nitrogen content. **(I)** Sucrose content. **(J)** Soluble sugar content. **(K)** Starch content. Data represented as means ± SD (*n* = 4). Different letters on the bars indicates significant difference by the one-way ANOVA and Duncan’s multiple range method (*P* < 0.05).

As shown in **Figure 1A**, *A. paniculata* is extremely sensitive to low temperature (LT). *A. paniculata* grown vigorously at 30°C, but its growth was inhibited and its development was accelerated when the temperature was 20°C and 25°C, in which conditions plants showed early flower branch differentiation and even early flowering. Plants cannot survive at 10°C, and the growth was arrested at 15°C.

Anthocyanin was remarkably accumulated when temperature was lower than 25°C (**Figure 1B**), while soluble protein was decreased gradually with the increase of temperature (**Figure 1C**). Additionally, LT remarkably increased the contents of H_2_O_2_ and MDA, as well as the activity of SOD (**Figures 1D–F**), while the CAT activity was dramatically reduced when the temperature was lower than 20°C (**Figure 1G**). These results suggested that *A. paniculata* is a LT-sensitive plant, which is damaged from LT lower than 25°C.

Leaf N content showed an upward tendency with the increase of temperature, but there was no significant difference among 20°C, 25°C, and 30°C (**Figure 1H**). Sucrose, soluble sugar and starch contents were reduced with the increase of temperature, but they were not different between 25°C and 30°C (**Figures 1I–K**). The results indicated that LT suppressed C and N metabolism of *A. paniculata*. In the following study, 20°C was represented as LT stress.

### 3.2 DH-Fe pretreatment alleviated LT stress induced oxidation stress

**Figure 2:**
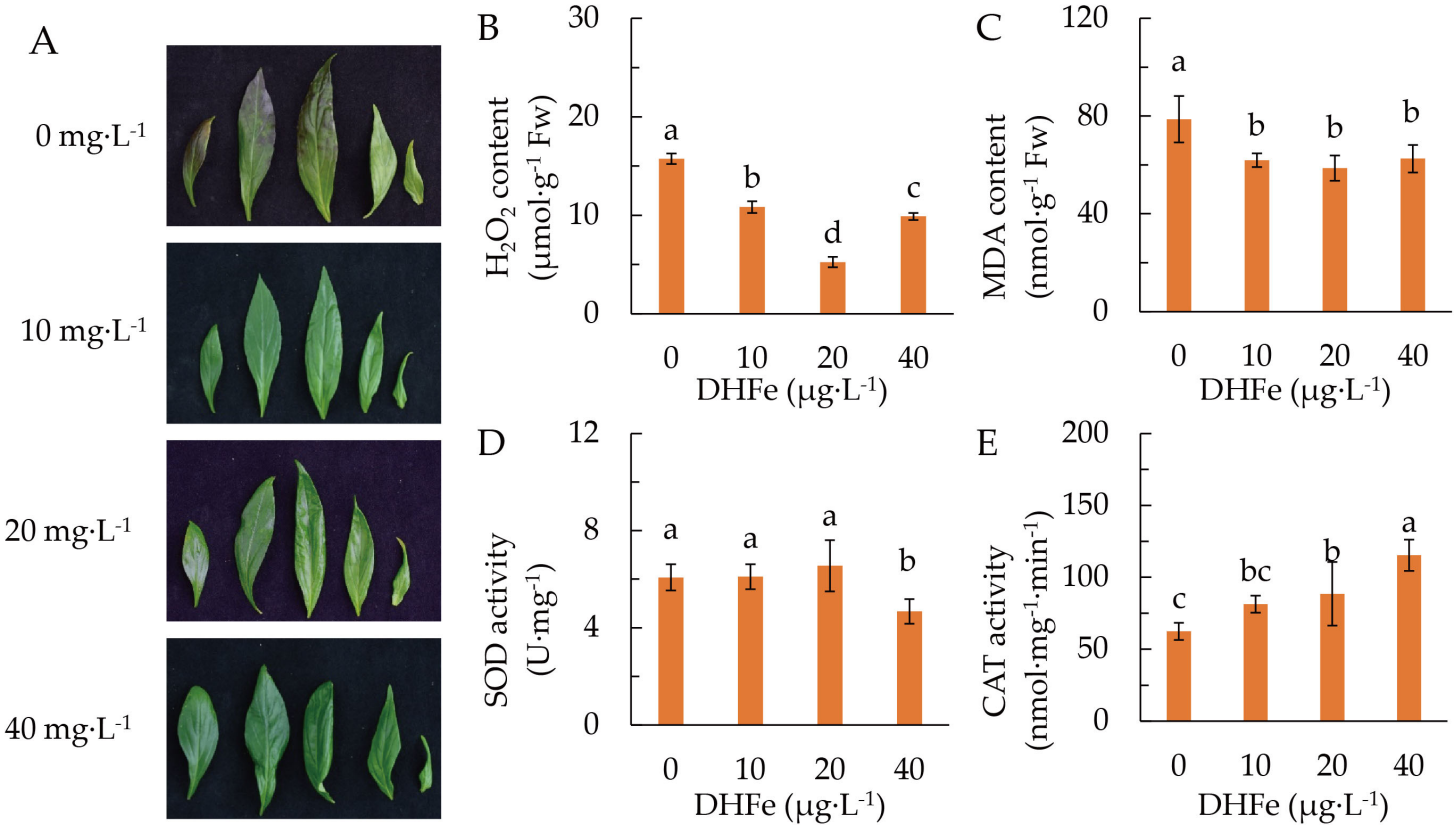
Effects of DH-Fe on antioxidant capacity. **(A)** Leaves phenotype was pictured 7 days after LT treatments. **(B)** Hydrogen peroxide content. **(C)** Malondialdehyde content. **(D)** Superoxide dismutase activity. **(E)** Catalase activity. Data represented as means ± SD (*n* = 4). Different letters on the bars indicates significant difference by the one-way ANOVA and Duncan’s multiple range method (*P* < 0.05).

Plants were pretreated with different DH-Fe concentrations and grown at 20°C. It was clear that application of DH-Fe reduced the accumulation of anthocyanin in leaves under LT stress (**Figure 2A**). The H_2_O_2_ and malondialdehyde (MDA) contents were remarkably reduced by DH-Fe, with the lowest contents at 20 μg·L^-1^ DH-Fe (**Figures 2B, C**). The SOD activity was significantly repressed when the DH-Fe concentration was 40 μg·L^-1^ (**Figure 2D**). In contrast, the CAT activity was triggered by DH-Fe application, with a significant increase at 20 and 40 μg·L^-1^ (**Figure 2E**). Apparently, DH-Fe is an effective protectant for plants under LT stress.

### 3.3 DH-Fe pretreatment reduced LT stress induced sugar accumulation

**Figure 3:**
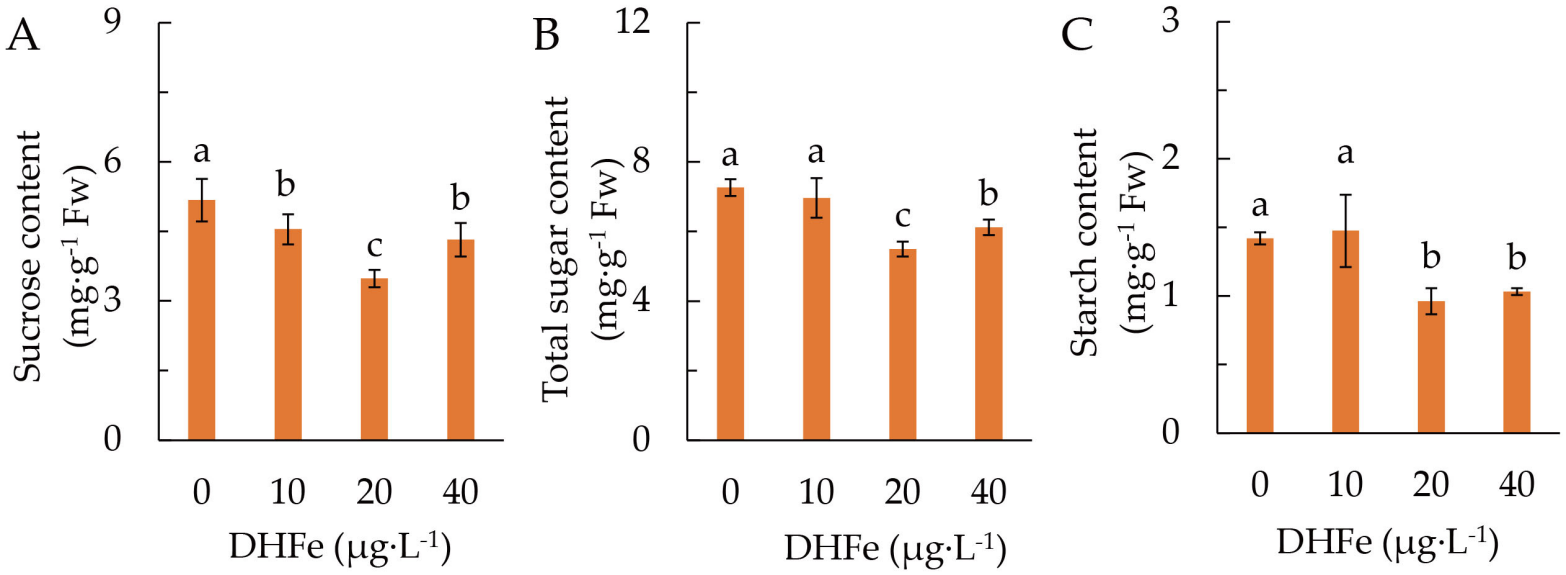
Effects of DH-Fe on sugar and starch accumulation. **(A)** Sucrose content. **(B)** Soluble sugar content. **(C)** Starch content. Data represented as means ± SD (*n* = 4). Different letters on the bars indicates significant difference by the one-way ANOVA and Duncan’s multiple range method (*P* < 0.05).

DH-Fe remarkably reduced the accumulation of sucrose, with the lowest content at 20 μg·L^-1^ (**Figure 3A**). Both total soluble sugar and starch contents were not different from the control at 10 μg·L^-1^ DH-Fe, but they were remarkably lower at 20 and 40 μg·L^-1^ DH-Fe (**Figures 3B, C**).

**Figure 4:**
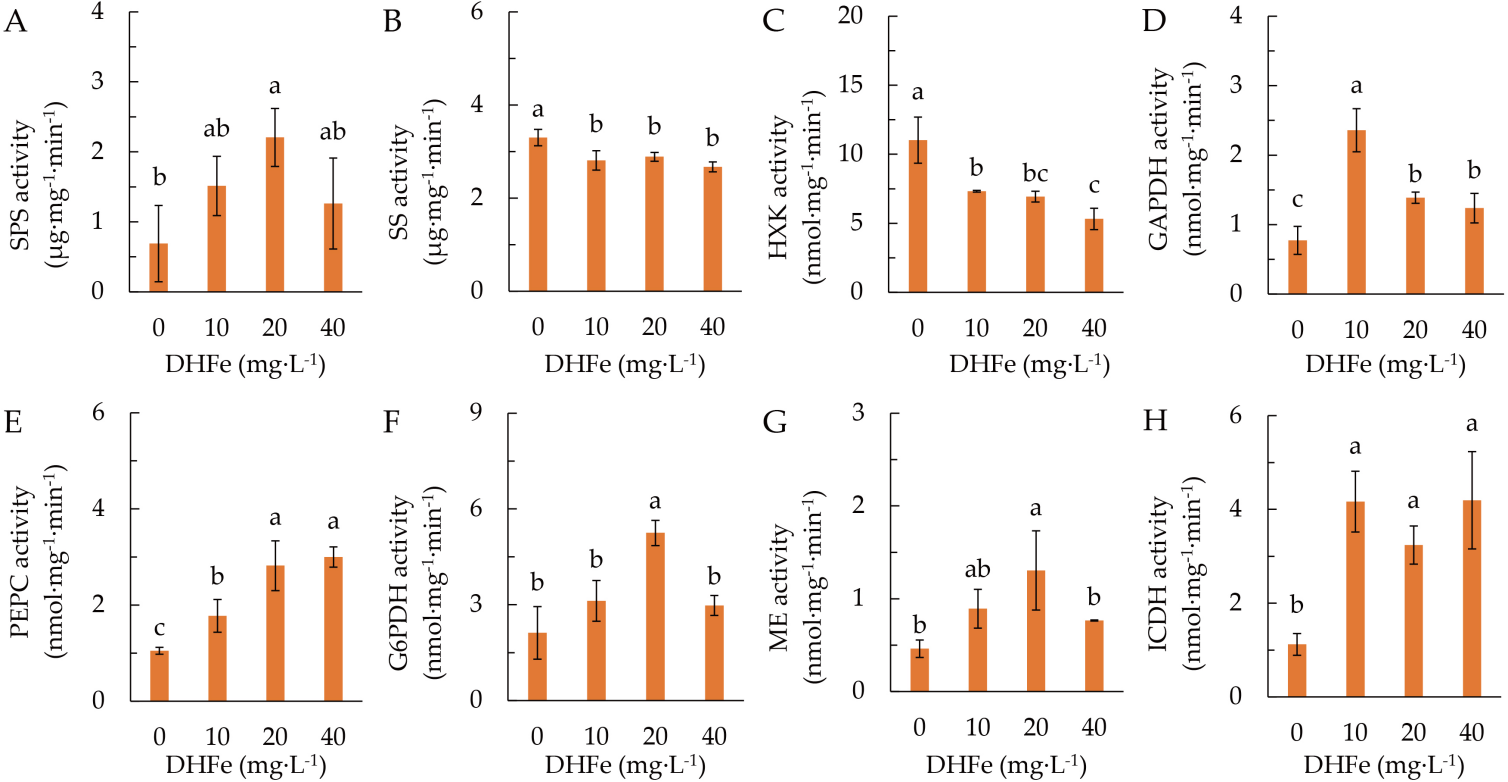
Effects of DH-Fe on carbon metabolism enzymes. **(A)** Sucrose phosphate synthase (SPS) activity. **(B)** Sucrose synthase (SS) activity. **(C)** Hexokinase (HXK) activity. **(D)** Glyceraldehyde 3-phosphate dehydrogenase (GAPDH) activity. **(E)** Phosphoenolpyruvate carboxylase (PEPC) activity. **(F)** Glucose-6-phosphate dehydrogenase (G6PDH) activity. **(G)** Malic enzyme (ME) activity. **(H)** Isocitrate dehydrogenase (ICDH) activity. Data represented as means ± SD (*n* = 3). Different letters on the bars indicates significant difference by the one-way ANOVA and Duncan’s multiple range method (*P* < 0.05).

The activity of SPS was remarkably higher at 20 μg·L^-1^ DH-Fe than that in the control (**Figure 4A**). In contrast, DH-Fe treatments significantly suppressed SS and HXK activities (**Figures 4B, C**). The GAPDH, PEPC and ICDH activities were remarkably increased by all DH-Fe concentrations (**Figures 4D, E, H**), while the G6PDH and ME activities were significantly higher at 20 μg·L^-1^ DH-Fe (**Figures 4F, G**). The results revealed that DH-Fe stimulated sugar catabolism in *A. paniculata* under LT stress.

### 3.4 DH-Fe pretreatment increased N metabolism under LT stress

**Figure 5:**
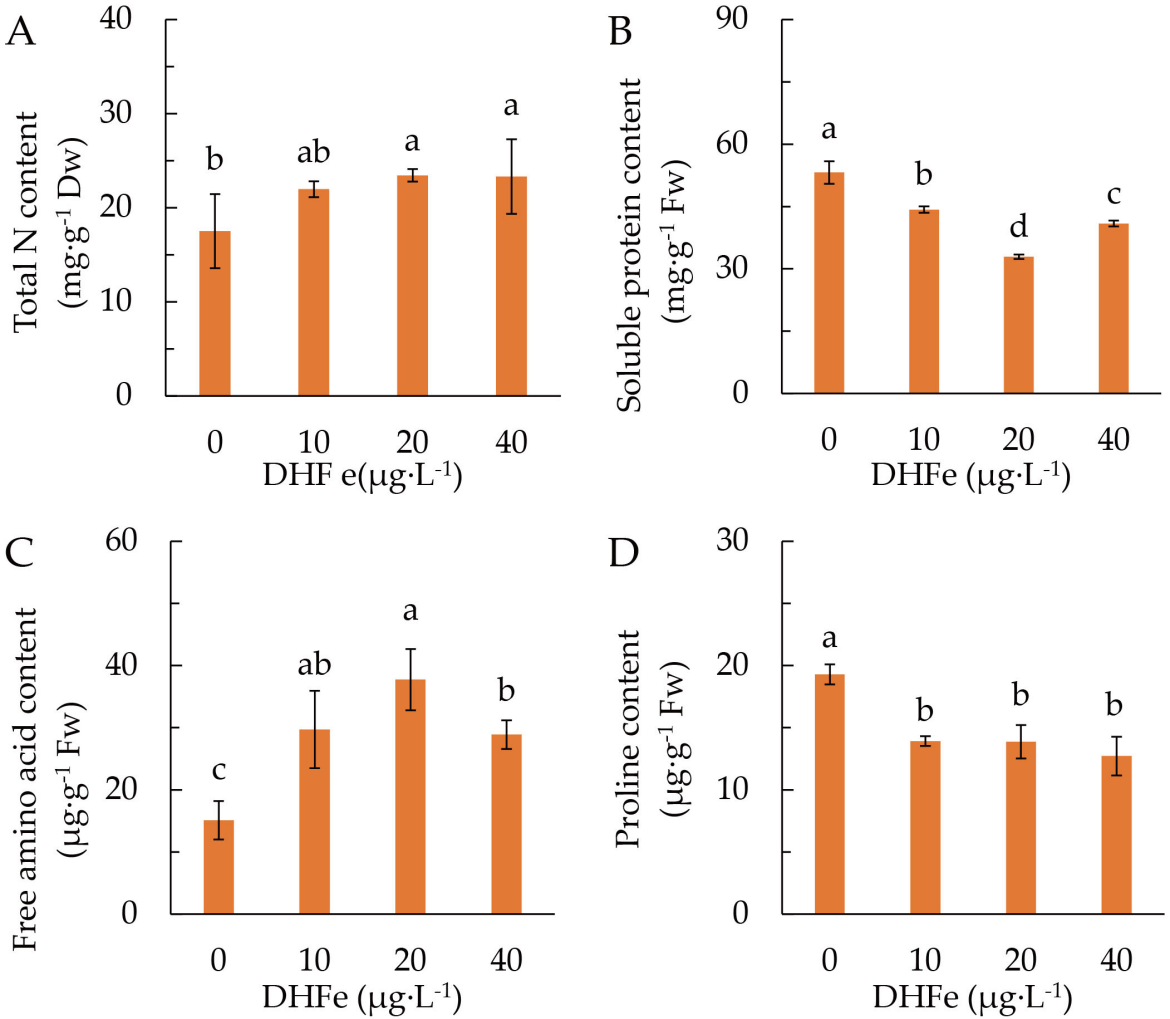
Effects of DH-Fe on N status in *A*. *paniculata* grown under low temperature. **(A)** Total N content. **(B)** Soluble protein content. **(C)** Free amino acid content. **(D)** Proline content. Data represented as means ± SD (*n* = 4). Different letters on the bars indicates significant difference by the one-way ANOVA and Duncan’s multiple range method (*P* < 0.05).

DH-Fe application raised the content of leaf total N, which was significantly higher at 20 and 40 μg·L^-1^ DH-Fe (**Figure 5A**). However, DH-Fe remarkably declined the accumulation of soluble protein, and the lowest soluble protein content was observed at 20 μg·L^-1^ DH-Fe (**Figure 5B**). In line with total N, free amino acids content was markedly enhanced by DH-Fe, with the greatest at 20 μg·L^-1^ DH-Fe (**Figure 5C**). Conversely, the proline content was descended in DH-Fe treatments (**Figure 5D**).

**Figure 6:**
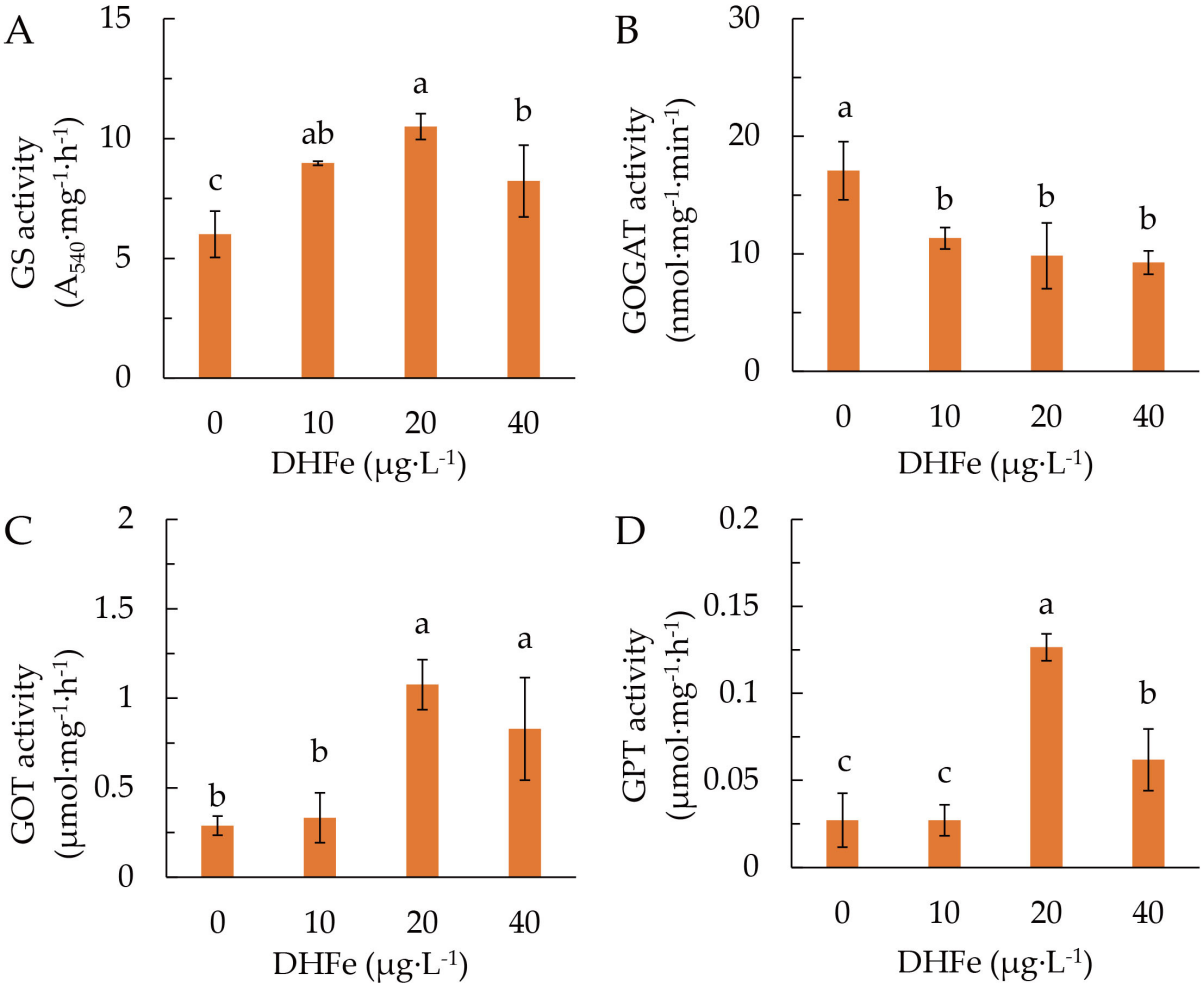
Effects of DH-Fe on N metabolic enzymes in *A*. *paniculata* grown under low temperature. **(A)** Glutamine synthase (GS) activity. **(B)** Glutamate synthase (GOGAT) activity. **(C)** Glutamic-oxaloacetic transaminase (GOT) activity. **(D)** Glutamic-pyruvic transaminase (GPT) activity. Data represented as means ± SD (*n* = 3). Different letters on the bars indicates significant difference by the one-way ANOVA and Duncan’s multiple range method (*P* < 0.05).

DH-Fe application considerably enhanced the GS activity (**Figure 6A**), but reduced the GOGAT activity (**Figure 6B**). Both GOT and GPT activities were remarkably increased at 20 and 40 μg·L^-1^ DH-Fe (**Figures 6C, D**). These results indicated that DH-Fe promoted N uptake and assimilation in *A. paniculata* under LT stress.

### 3.5 DH-Fe pretreatment decreased andrographolide accumulation under LT stress

**Figure 7:**
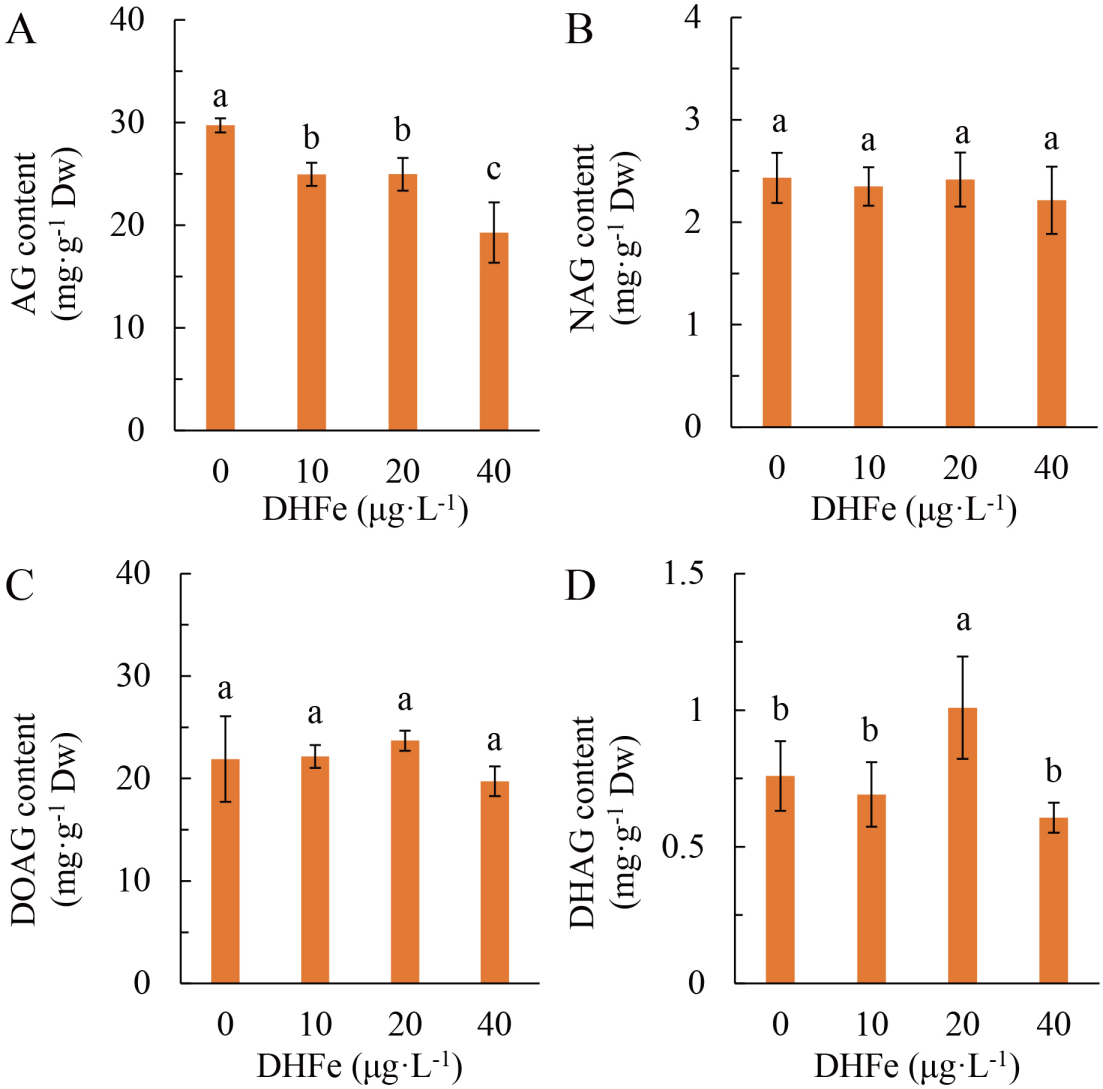
Effects of DH-Fe on diterpene lactones contents in *A*. *paniculata* grown under low temperature. **(A)** Andrographolide (AG) content. **(B)** Neoandrographolide (NAG) content. **(C)** 14-Deoxyandrographolide (DOAG) content. **(D)** 14-Deoxy-11,12-didehydroandrographolide (DHAG) content. Data represented as means ± SD (*n* = 4). Different letters on the bars indicates significant difference by the one-way ANOVA and Duncan’s multiple range method (*P* < 0.05).

DH-Fe pretreatment mainly affected andrographolide (AG) of diterpene lactones in *A. paniculata*. With the growing DH-Fe concentration, AG content was decreased gradually and significantly (**Figure 7A**). However, the contents of neoandrographolide (NAG) and 14-deoxyandrographolide (DOAG) were not significantly affected by DH-Fe (**Figures 7B, C**). In contrast, 20 μg·L^-1^ DH-Fe remarkably increased the content of 14-deoxy-11, 12-didehydroandrographolide (DHAG) (**Figure 7D**). The AG content was negatively correlated with N content (*P* < 0.05) but positively correlated with sugars contents (*P* > 0.05) (**Table 1**).

**Table 1:** Correlation analysis of the contents of diterpenoid lactones and N and carbohydrates.

Index	AG	NAG	DOAG	DHAG
Total N	-0.623* (0.030)	-0.541 (0.069)	-0.319 (0.312)	0.162 (0.616)
Sucrose	0.333 (0.266)	-0.046 (0.866)	0.027 (0.920)	-0.070 (0.798)
Soluble sugar	0.396 (0.181)	0.096 (0.723)	0.140 (0.606)	-0.012 (0.965)
Starch	0.434 (0.139)	0.210 (0.436)	0.102 (0.706)	-0.075 (0.783)

The correlation between two variables was expressed by the Pearson correlation coefficient. The values in brackets were *P* values. Asterisk (*) indicates significant correlation at *P* < 0.05 level. AG, andrographolide; NAG, neoandrographolide; DOAG, 14-deoxyandrographolide; DHAG, dehydroandrographolide.

## 4 Discussion

*A. paniculata* originated from South Asia and is widely distributed in South Asia, Southeast Asia, and southern China. Low temperature is the main obstacle it faces during its northward introduction. In this study, by treating with temperature gradients, we found that *A. paniculata* is very sensitive to low temperature. Temperature at 20°C-25°C not only inhibited its growth but also accelerated its development, and temperature lower than 15°C retarded its growth or even lethal for the plant. Thus, 15°C and 25°C could be the temperature thresholds for the survival and vigorous growth of this plant species, respectively. To reveal the physiological mechanism by which *A. paniculata* in response to LT is of great significance for enhancing its LT tolerance via application of exogenous regulators and breeding.

LT stress dysregulates many physiological processes in plant such as reactive oxygen species (ROS) metabolism, photosynthesis, respiration, and N metabolism (Airaki et al., 2012). LT evokes the production of ROS in plants, predisposing them to oxidative bursts and stress within plant cells. The production and scavenging of ROS are pivotal for plants to adapt to LT stress. LT conditions (e.g. 15°C, 20°C, and 25°C) elevated oxidative stress of plants as indicated by increased H_2_O_2_ and MDA contents, as well as enhanced SOD activity. Under LT conditions, limitation of CO_2_ fixation coupled with over-reduction of the electron transport chain is the main cause of ROS production (Suzuki and Mittler, 2006). Dihydroporphyrin is a type of reduced porphyrin, a specific chlorophyll derivative; Fe is an important factor mediating chlorophyll biosynthesis. Foliar DH-Fe application restores leaf chlorophyll content, enhances the activities of antioxidant enzymes, and decreases the generation rate of H_2_O_2_ (Cao et al., 2016). Here, DH-Fe at 20 and 40 μg·L^-1^ considerably dropped the accumulation of H_2_O_2_ and MDA, but remarkably increased the CAT activity under LT stress, suggesting that appropriate concentration of DH-Fe alleviates LT-induced oxidative stress by enhancing CAT activity in *A. paniculata* plants.

Sugars function as osmoprotectants, whose accumulation has been considered to be an adaptive mechanism of plants to stresses (Ahmad et al., 2020). However, high accumulation of sugars leads to plant growth retardation and even is toxic to plants (Huang et al., 2022). Excessive accumulation of sugars triggers the synthesis of anthocyanin (Murakami et al., 2008). Here, we found that LT stress induced a great accumulation of anthocyanin in leaves, which was associated with the build-up of sugars. It was suggested that sugar could inhibit plant growth via the synthesis of anthocyanin. An extensive accumulation of anthocyanin coupled with the increases in SOD activity and MDA content lead to plant senescence (Jakhar and Mukherjee, 2014). The increased anthocyanin content and oxidative stress under LT stress could contribute to LT-induced early flowering in *A. paniculata*. Thus, the catabolism of sugars (carbohydrates) could be beneficial for plants to acclimate LT stress. DH-Fe alleviates plant senescence under stress condition (Chu et al., 2023). The results in this study showed that high DH-Fe concentration eliminated the phenotype of anthocyanin accumulation, accompanying with reduced sugar accumulation. Simultaneously, the SOD activity and MDA content were decreased. The results indicated that DH-Fe delayed senescence of *A. paniculata* plants under LT stress by accelerating sugar catabolism.

C partitioning between biosynthetic and dissimilatory pathways is essential in plant LT acclimation (Kitashova et al., 2023). Several enzymes such as sucrose phosphate synthase (SPS) and phosphoenolpyruvate carboxylase (PEPC) play important roles in mediating C metabolic flow into downstream TCA cycle and N assimilation (Champigny and Foyer, 1992; Yu et al., 2017; Li et al., 2018b). The pentose phosphate pathway (PPP) is important to produce precursors for nucleotide and amino acid biosynthesis, to provide reductant for anabolism, and to defeat oxidative stress (Stincone et al., 2014). In this study, the activities of SPS, PEPC, G6PDH and GAPDH were elevated by pretreatment of DH-Fe under LT stress, whereas the HXK and SS activities were downregulated. It was implied that DH-Fe pretreatment activated the PPP and downstream glycolysis under LT stress.

NADPH oxidase contributes to the production of ROS in LT conditions (Suzuki and Mittler, 2006). It has been proposed that the NADPH-generating dehydrogenases function in plant cold stress acclimation through their effect on the redox state of plant cells (Airaki et al., 2012). The PPP is a critical process producing NADPH, which functions in maintaining redox balance under stress situations. The key enzyme in PPP, NDAP-dependent G6PDH, together with other NADPH-generating enzymes such as ICDH and ME could provide NADPH for ATP synthesis (Lawlor and Tezara, 2009), N assimilation (Thormählen et al., 2015), and reduction of oxidized glutathione (Lascano et al., 1999). In the current study, the activities of G6PDH, ICDH and ME were remarkably increased by DH-Fe, implying that DH-Fe enhanced LT stress tolerance of *A. paniculata* plants by promoting the production of reductants.

Increase in sugar catabolism not only maintains cell redox homeostasis but also provides C skeletons for N metabolism. The increased ICDH activity could provide more C skeletons (α-ketoglutarate) for N assimilation. In this study, DH-Fe reversed the inhibition of LT stress on N metabolism, which was corroborated by the increases in the activities of GS, GOT and GTP and the content of N. It was suggested that DH-Fe promotes N uptake and assimilation to enhance the resistance of *A. paniculata* to LT stress. N metabolism has been demonstrated to be essential for plant fitness (Wang and Lü, 2020), although it itself is one of the main processes that are susceptible to adversities in plants. There are several mechanisms that N metabolism involves in plant resistance to adverse circumstances, including dissipating excessive energy by assimilation of inorganic N (Yi et al., 2014), stabilizing protein and maintaining photosynthesis (Zhong et al., 2019), providing osmolytes such as soluble protein, free amino acids and proline (Ashraf and Foolad, 2007), and improving cell redox homeostasis (Soualiou et al., 2023). We found that soluble protein and proline contents were reduced by DH-Fe application under LT stress, which was similar to the results those under optimal temperature (30°C). The findings illustrated that the enhanced N metabolism resulted from DH-Fe application is less likely to alleviate the damage of LT stress to *A. paniculata* by enhancing osmotic regulation. Alternatively, it could involve in dissipating excessive energy and maintaining cell redox homeostasis. Fundamentally, energy excess and redox imbalance are the main issues that plants face under LT stress.

Secondary metabolites are important bioactive ingredients in medicinal plants. They also act as defensive substances enhancing plant stress resistance. Under well growth conditions, in which N metabolism activity is vigorous and sugar accumulation is relatively low, the accumulation of secondary metabolites is depressed (Rühmann et al., 2002). It has been proposed that the synthesis of protein and amino acids required for plant growth could attenuate the allocation of C metabolic flow to the synthesis of secondary metabolites (Larbat et al., 2016). Diterpene lactones are the major bioactive secondary metabolites in *A. paniculata*. Their accumulation is induced by several adversities such as drought (Chen et al., 2020), salt stress (Talei et al., 2015), and ultraviolet radiation (Sun et al., 2022). Andrographolide content was decreased with the increase of DH-Fe concentration, which was in line with the decrease of sugar contents but opposite to the increase of N content. The results reflected that DH-Fe ameliorated plant growth under LT stress and therefore, diminished the allocation of carbohydrates to andrographolide biosynthesis in *A. paniculata*.

## 5 Conclusion

**Figure 8:**
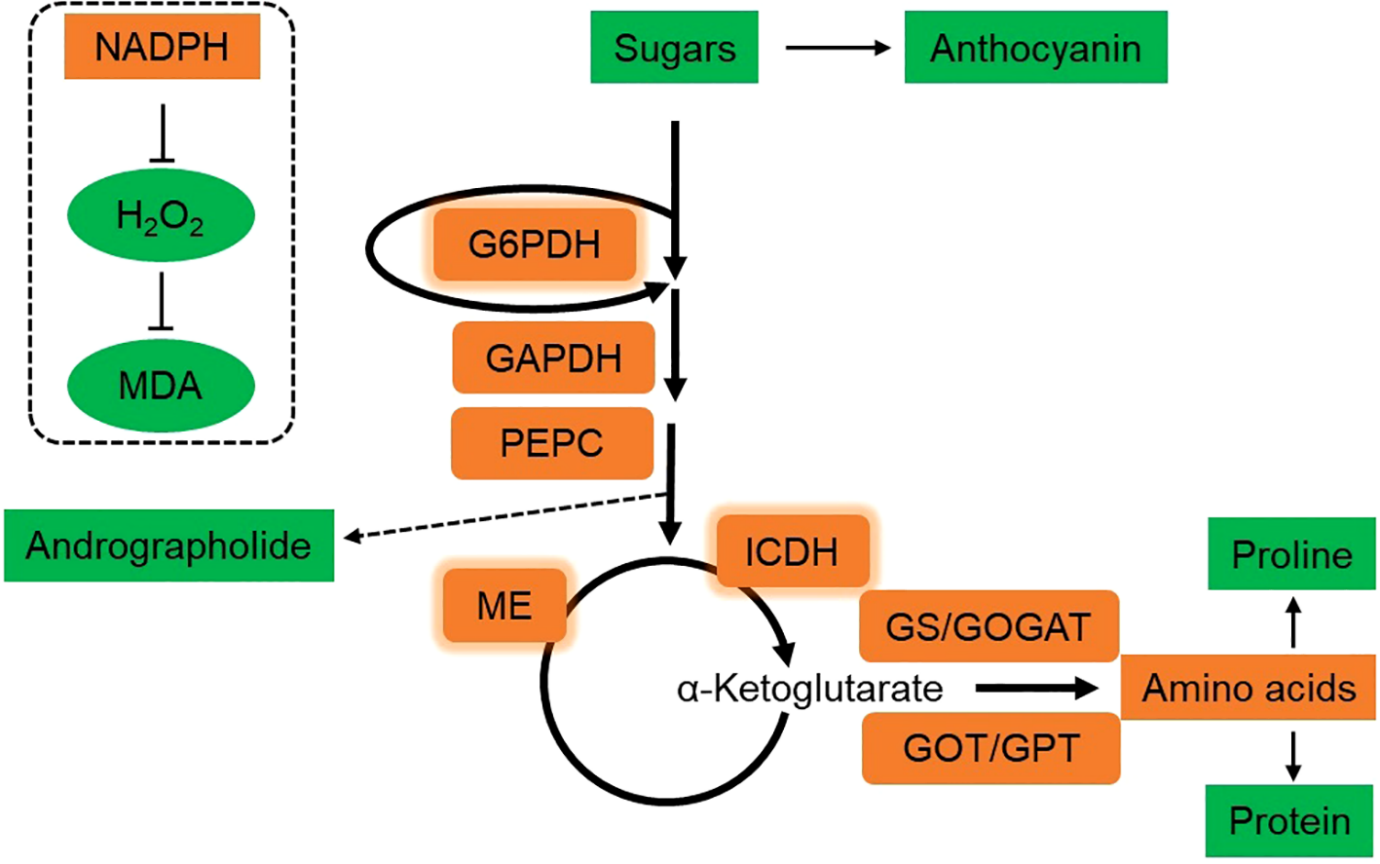
Schematic diagram of the mechanism of DH-Fe enhancing the LT adaptability of *A. paniculata*. The brown box represents enhanced, and green box represents decreased by DH-Fe. The thickness of the arrows indicates the relative magnitude of metabolic flux. Under LT conditions, DH-Fe reduced the accumulation of sugars and anthocyanin, but increased the catabolism of sugars. The activities of G6PDH, GAPDH, PEPC, ME, and ICDH were enhanced by DH-Fe, resulting in increased allocation of C metabolic flow towards to N assimilation and amino acids synthesis. Simultaneously, andrographolide content was decreased. G6PDH, ME, and ICDH are NADPH-generating enzymes. The increase of their activities could lead to high production of NADPH, which can prevent excessive accumulation of H_2_O_2_ and alleviate lipid peroxidation.

*A. paniculata* is a low temperature sensitive plant with a threshold of 25°C. Application of DH-Fe alleviated LT-induced oxidative stress in *A. paniculata* plants by increasing antioxidant capacity, with the most effective concentration at 20 μg·L^-1^. The role of DH-Fe in inducing osmotic regulation was excluded due to reduced soluble protein, proline and sugars contents. DH-Fe promoted sugar catabolism via the enzymes of G6PDH, GAPDH, PEPC, ME, and ICDH, which provided reductant for redox homeostasis and C skeletons for downstream N assimilation (**Figure 8**). Thus, C and N metabolisms play the center role in DH-Fe attenuating LT stress induced oxidative stress. The results indicated that application of DH-Fe is an effective strategy for *A. paniculata* plants overcoming LT stress. However, a long-term experiment is needed to evaluate the effect of DH-Fe on improving the growth of *A. paniculata* under LT conditions.

## Data Availability

The original contributions presented in the study are included in the article/supplementary material. Further inquiries can be directed to the corresponding author/s.
